# Quackery

**Published:** 1836

**Authors:** 


### Art. II.—Quackery.

The following we extract from the above named No. of the Boston Medical and Surgical Journal.

" *Botanic Physicians. Supreme Court.*—Barent P. Staats, vs. John Thomson.—This was an action brought by B. P. Staats, President of the Albany county Medical Society, against John Thomson, a Botanic Physician, for practicing medicine contrary to the laws of this State, (New York). Defendant produced a patent from the United States to his father for the exclusive right and use of botanic medicines, and he, as *assignee* of the patent, claimed a right under said patent to practice and prescribe for patients. The Justice's Court of the city of Albany decided that he, *Thomson*, had no right to practice medicine for fee or *reward,* unless he has a diploma from some regular incorporated or medical school or society, and fined him the sum of $25; from which decision Thomson appealed to the *Supreme* Court. The Supreme Court affirmed the decision; consequently Botanic Physicians have no right to recover for services in the State of New York, nor, is it believed, in any other. It is to be hoped that some one, possessing the energy of our friend Dr. Staats, will ascertain the right and privileges of steam quacks to pepper mankind to death in the respectable old Commonwealth of Massachusetts, the head quarters of the originator of the lobelia cascading system."

